# Reverse genetic screen reveals that Il34 facilitates yolk sac macrophage distribution and seeding of the brain

**DOI:** 10.1242/dmm.037762

**Published:** 2019-03-08

**Authors:** Laura E. Kuil, Nynke Oosterhof, Samuël N. Geurts, Herma C. van der Linde, Erik Meijering, Tjakko J. van Ham

**Affiliations:** 1 Department of Clinical Genetics, Erasmus University Medical Center, Wytemaweg 80, 3015 CN Rotterdam, The Netherlands; 2 European Research Institute for the Biology of Ageing, University Medical Center Groningen, Antonius Deusinglaan 1, 9713 AV Groningen, The Netherlands; 3 Biomedical Imaging Group Rotterdam, Departments of Medical Informatics and Radiology, Erasmus University Medical Center, Wytemaweg 80, 3015 CN Rotterdam, The Netherlands; 4 Quantitative Imaging, Faculty of Applied Sciences, Delft University of Technology, Lorentzweg 1, 2628 CJ Delft, The Netherlands

**Keywords:** Reverse genetic screen, Microglia, Brain development, Macrophages, Hematopoiesis

## Abstract

Microglia are brain-resident macrophages, which have specialized functions important in brain development and in disease. They colonize the brain in early embryonic stages, but few factors that drive the migration of yolk sac macrophages (YSMs) into the embryonic brain, or regulate their acquisition of specialized properties, are currently known. Here, we present a CRISPR/Cas9-based *in vivo* reverse genetic screening pipeline to identify new microglia regulators using zebrafish. Zebrafish larvae are particularly suitable due to their external development, transparency and conserved microglia features. We targeted putative microglia regulators, by Cas9/gRNA complex injections, followed by Neutral-Red-based visualization of microglia. Microglia were quantified automatically in 3-day-old larvae using a software tool we called SpotNGlia. We identified that loss of zebrafish colony-stimulating factor 1 receptor (Csf1r) ligand, Il34, caused reduced microglia numbers. Previous studies on the role of IL34 in microglia development *in vivo* were ambiguous. Our data, and a concurrent paper, show that, in zebrafish, *il34* is required during the earliest seeding of the brain by microglia. Our data also indicate that Il34 is required for YSM distribution to other organs. Disruption of the other Csf1r ligand, Csf1, did not reduce microglia numbers in mutants, whereas overexpression increased the number of microglia. This shows that Csf1 can influence microglia numbers, but might not be essential for the early seeding of the brain. In all, we identified *il34* as a modifier of microglia colonization, by affecting distribution of YSMs to target organs, validating our reverse genetic screening pipeline in zebrafish.

This article has an associated First Person interview with the joint first authors of the paper.

## Abstract (precis)

**Summary:** Using a new reverse genetic screening method for zebrafish microglia development, involving automated image analysis, we identified that *il34* facilitates yolk sac macrophage distribution to target organs, including the brain.

## INTRODUCTION

Tissue macrophages, in addition to their immunological roles, modulate organogenesis and exhibit organ-specific regulatory properties that are thought to affect virtually all organs in vertebrates (Gordon and Martinez-Pomares, 2017; Pollard, 2009). Microglia are the brain's resident macrophages, and have roles in brain development and homeostasis. Described functions of microglia include the removal of dead cells and debris, modulation of neuronal connectivity by synaptic pruning and maintenance of myelin-producing cells (Colonna and Butovsky, 2017; Hagemeyer et al., 2017; Salter and Stevens, 2017; Thion and Garel, 2017). Defects in microglia function have been implicated in neurodevelopmental disorders such as autism spectrum disorder (Salter and Stevens, 2017). Pathogenic variants in genes thought to primarily affect microglia cause rare white matter disorders, including Nasu-Hakola disease and adult-onset leukoencephalopathy with axonal spheroids (ALSP), which may be caused by loss of microglia activity (Oosterhof et al., 2018; Paloneva et al., 2002; Rademakers et al., 2011; Sundal et al., 2012). In line with this, there is accumulating evidence that replenishing brain myeloid cells by hematopoietic cell transplantation has powerful therapeutic potential in leukodystrophy and metabolic diseases affecting the brain, and better understanding the molecular regulation of brain colonization by microglia could lead to ways to facilitate this (Eichler et al., 2016; Stepien et al., 2017; van Rappard et al., 2016). However, the exact genes and mechanisms underlying the emergence of microglia in the brain and acquisition of their functional properties are still poorly understood.

Microglia originate from macrophage progenitors in the embryonic yolk sac, known as yolk sac macrophages (YSMs), which colonize the brain during early embryonic development (Ginhoux et al., 2010; Herbomel et al., 2001). Once they arrive in the brain, they acquire a highly ramified morphology, proliferate extensively and form a brain-wide network with non-overlapping territories (Svahn et al., 2013). The transition from YSM to mature microglia or other tissue-resident macrophages involves several differentiation stages characterized by distinct transcriptional profiles (Mass et al., 2016; Matcovitch-Natan et al., 2016). The progression through these transcriptional states is synchronized with, and most likely driven by, the different stages of brain development, as microglia gene expression is highly sensitive to changes in the microenvironment and tissue macrophage identity is mostly determined by the host environment (Gosselin et al., 2014; Lavin et al., 2014; Matcovitch-Natan et al., 2016; Thion et al., 2017). For the majority of the genes specifically expressed in microglia, the function is still unknown, and as many of these genes are rapidly downregulated when they are taken out of the brain, it is difficult to study their functions *in vitro* (Beutner et al., 2013; Gosselin et al., 2017). In mammals, microglia development is relatively inaccessible to study, as YSMs emerge during development *in utero*. Despite progress in identifying methods to recreate microglia-like cells *in vitro*, improved understanding of their ontogeny is needed to guide *in vitro* efforts (Lee et al., 2018; Muffat et al., 2016). Therefore, identification of the functions of genes affecting microglia development could provide valuable insights into regulation of microglia development and function *in vivo*.

Zebrafish embryos are relatively small and transparent, relatively easy to manipulate genetically and develop *ex utero*, which makes them highly suitable for *in vivo* genetic studies (Oosterhof et al., 2015). We recently showed that microglia gene expression is well conserved between zebrafish and mammals and that, as shown in mice, loss of the two zebrafish homologs of the colony-stimulating factor 1 receptor (Csf1ra and Csf1rb) leads to absence of microglia (Dai et al., 2002; Erblich et al., 2011; Oosterhof et al., 2017, 2018). Phenotype-driven, forward genetic screens in zebrafish have identified several microglia mutants with a defect in microglia development or function. Processes affected in these mutants include hematopoiesis, regulation of inflammation, phosphate transport and lysosomal regulation, which implies that these various processes are all critical for microglia development and function (Demy et al., 2017; Meireles et al., 2014; Rossi et al., 2015b; Shen et al., 2016; Shiau et al., 2013). However, such forward genetic screens are laborious and relatively low throughput. A candidate-driven reverse genetic screening approach could lead to the identification of additional genes important for microglia. The CRISPR/Cas9-system can be used to create insertions or deletions (indels) in target genes via the repair of Cas9-induced double-strand breaks by error-prone non-homologous end joining (NHEJ) (Cong et al., 2013). Injection of gene-specific guide RNAs (gRNAs) and Cas9 mRNA can lead to gene disruption sufficiently effective to allow small-scale reverse genetic screening; for example, to identify new genes involved in electrical synapse formation (Shah et al., 2015). Alternatively, active Cas9-gRNA ribonucleoprotein complexes (RNPs) injected into fertilized zebrafish oocytes can more efficiently induce indels in target genes, and the resulting genetic mosaic zebrafish can phenocopy existing loss-of-function mutants (CRISPants) (Burger et al., 2016; Hwang et al., 2013).

Here, we present a scalable CRISPR/Cas9-based reverse genetic screening pipeline in zebrafish to identify important genetic microglia regulators using zebrafish. In zebrafish larvae, microglia can be visualized by the vital dye Neutral Red (NR), which shows a more pronounced staining in microglia over other macrophages and has been used as an effective readout for microglia numbers in forward genetic screens (Herbomel et al., 2001; Meireles et al., 2014; Shen et al., 2016; Shiau et al., 2013). We developed an image quantification tool, SpotNGlia, to automatically detect the brain boundaries and count NR^+^ microglia. Of the 20 putative microglia regulators we targeted by CRISPR/Cas9-mediated reverse genetics, disruption of *interleukin 34* (*il34*) showed the strongest reduction in microglia numbers in developing zebrafish larvae. In mammals, IL34 is one of two ligands of the microglia regulator CSF1R. Further analysis in stable *il34* mutants revealed that *il34* is mainly important for the recruitment of microglia to the brain, and likely other tissue resident macrophage populations, including Langerhans cells (LCs), to their target organs. Thus, we here present a scalable reverse genetic screening pipeline to identify additional new regulators important for microglia development and function.

## RESULTS

### CRISPants phenocopy existing mutants with microglia developmental defects

**Fig. 1.**
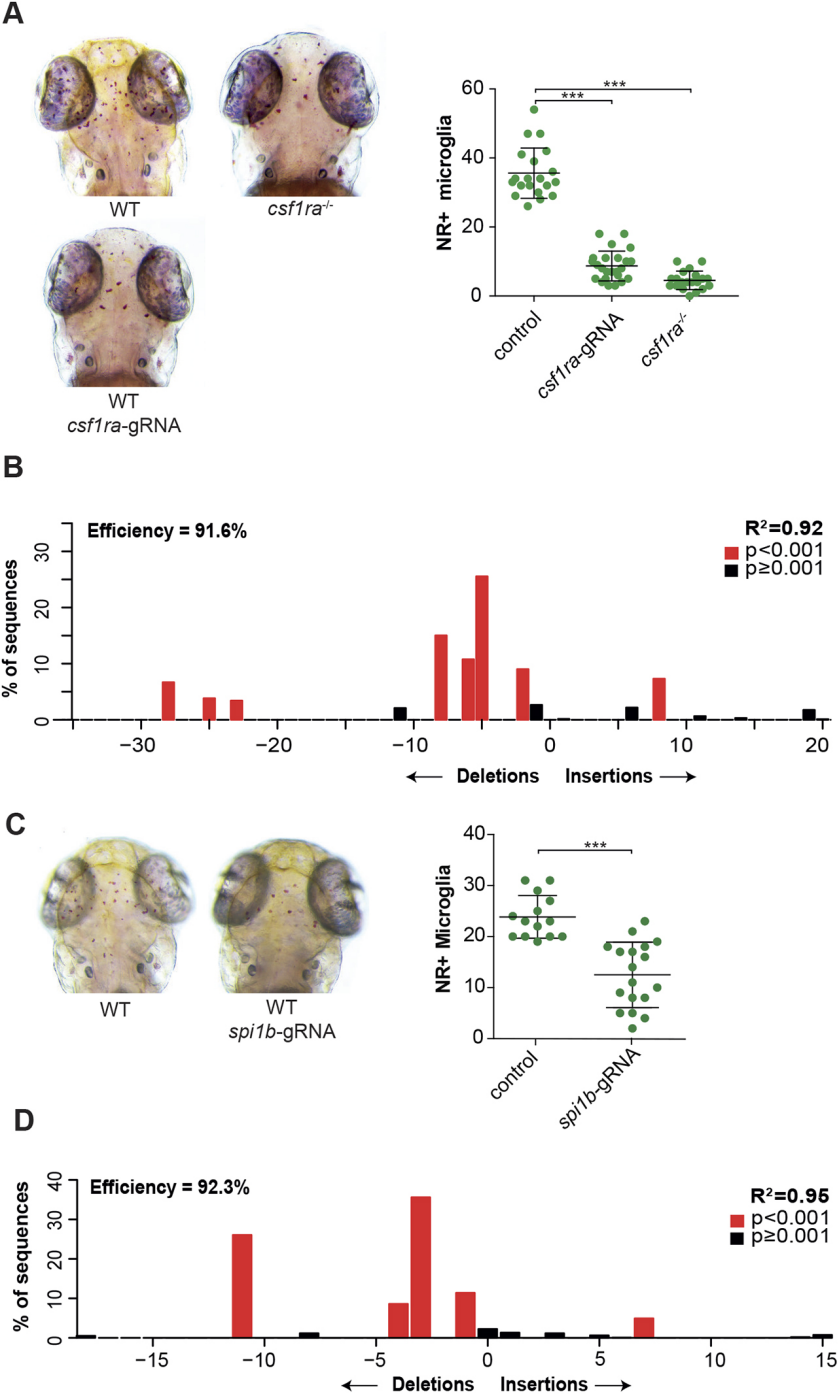
***csf1r* CRISPants phenocopy existing *csf1r* microglia mutants.** (A) Neutral Red (NR^+^) images and quantification of wild-type (WT), *csf1ra*^−/−^ and *csf1ra* CRISPant zebrafish larvae at 3 dpf. (B) Indel spectrum of a pool of *csf1ra* CRISPants calculated by TIDE. (C) NR images and quantification of WT and *spi1b* CRISPant zebrafish larvae at 3 dpf. (D) Indel spectrum of a representative individual *spi1b* CRISPant calculated by TIDE. The R^2^ value represents reliability of the indel spectrum. ***P<0.001. One-way ANOVA and Student's *t-*test. Each dot represents one larva. Error bars represent s.d.

Loss of one of several key macrophage regulators, including *Spi1* (encoding PU.1), *Irf8* and *Csf1r*, and their zebrafish homologs *spi1b* (Pu.1), *csf1ra* and *csf1rb*, and *irf8*, leads to defects in microglia development (Erblich et al., 2011; Herbomel et al., 2001; Horiuchi et al., 2012; Kierdorf et al., 2013; Rhodes et al., 2005; Shiau et al., 2015; Su et al., 2007). To investigate whether Cas9-gRNA RNPs targeting these regulators can be used to induce mutant microglia phenotypes directly, we injected zebrafish oocytes with RNPs targeting either *csf1ra* or *spi1b.* To assess whether CRISPR/Cas9-based targeting of those genes affects microglia development, we determined microglia numbers by NR staining at 3 days post-fertilization (dpf). At this time point, microglia have just colonized the optic tectum, are highly phagocytic and have low proliferative activity, which makes it an ideal time point to identify genes required for the earliest steps of microglia development (Herbomel et al., 2001; Xu et al., 2016). We quantified NR^+^ microglia in *csf1ra* CRISPants, in controls and in *csf1ra* loss-of-function mutants found in an N-ethyl-N-nitrosourea (ENU) mutagenic screen (hereafter called *csf1ra*^−/−^) (Parichy et al., 2000). Similar to *csf1ra^−/−^* mutants, *csf1ra* CRISPants showed an 80% reduction in the number of NR^+^ microglia compared with controls, suggesting highly effective targeting in F0 injected embryos (Fig. 1A). To assess the targeting efficiency of the *csf1ra* gene, we performed Sanger sequencing of the targeted locus of a small pool of *csf1ra* CRISPants, and calculated the spectrum and frequency of indels in the *csf1ra* gene using ‘tracking indels by decomposition’ (TIDE) software (Brinkman et al., 2014). The mutagenic efficiency was >90%, showing efficient mutagenesis (Fig. 1B). Similarly, *spi1b* CRISPants showed a strong reduction in the number of microglia and 65-95% mutagenic efficiency (Fig. 1C,D). This shows that CRISPR/Cas9-based mutagenesis can be used to reproduce mutant microglia phenotypes in Cas9-gRNA-RNP-injected zebrafish larvae.

### SpotNGlia semi-automatically counts microglia numbers

**Fig. 2.**
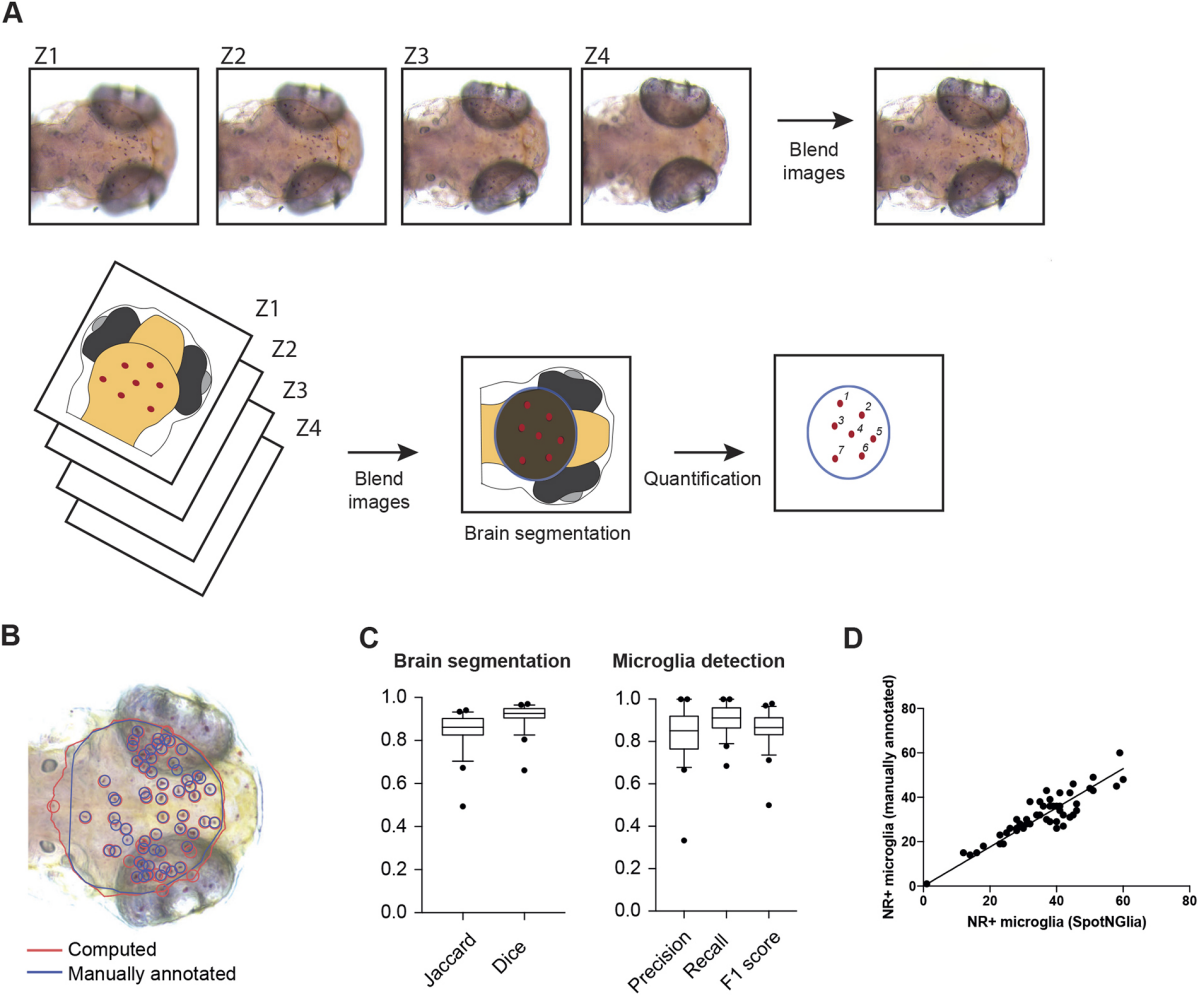
**SpotNGlia semi-automatically counts microglia numbers.** (A) Examples of *z*-stack images of NR-stained larvae and a schematic representation of the SpotNGlia analysis pipeline. (B) SpotNGlia output of test dataset with both manual (blue) and automated (red) brain segmentation and NR^+^ microglia annotation. (C) Box plots showing Jaccard and Dice indices for accuracy of brain segmentation and F1, precision and recall scores for the accuracy of NR^+^ microglia annotation. This is a Tukey boxplot: it uses the median and interquartile range (IQR) for the box. The whiskers extend to the most extreme data within 1.5×IQR. Data outside 1.5×IQR are considered outliers. (D) Correlation between manually and automated microglia quantification after manual correction for segmented brain area. Error bars represent s.d.

Manual quantification of NR^+^ microglia, across *z*-stack images, is time consuming and can be subjective. To standardize and speed up quantification, we developed a software tool, SpotNGlia, that automatically counts NR^+^ microglia in the optic tectum, where most microglia are located at 3 dpf. The SpotNGlia tool aligns stacked images of stained zebrafish larvae taken at different axial positions and blends the images into a single 2D image in which all NR^+^ cells are in focus (Fig. 2A). Next, the images are segmented by using polar transformation and dynamic programming to identify the edges of the optic tectum. Finally, NR^+^ cells are detected and counted by a spot detection technique based on multi-scale wavelet products (Olivo-Marin, 2002). To test the SpotNGlia software tool, we created and manually annotated a dataset with representative *z*-stack images of 50 NR-stained zebrafish larvae. To assess the accuracy of brain segmentation, Jaccard and Dice indices were determined, revealing indices of 0.86 (Jaccard) and 0.93 (Dice) (Fig. 2B,C). To assess the accuracy of microglia detection, we determined the precision, recall and F1 scores of the computed annotation, resulting in average scores of 0.85, 0.91 and 0.87, respectively (Fig. 2B-D). These results indicate that SpotNGlia is able to automatically identify the boundaries of the midbrain region, and the microglia within that region, in the vast majority of cases. To correct manually for those instances in which brain segmentation and microglia detection were not completely accurate, as determined by visual inspection, our tool offers the possibility of post hoc correction. In our experiments, we have found that SpotNGlia results in ∼80% reduction in the time it takes to quantify NR^+^ microglia numbers. In all, this indicates that SpotNGlia is a powerful tool for fast quantification of NR^+^ microglia numbers to assist in identifying novel genes important for generation of functional microglia.

### Reverse genetic screen reveals zebrafish Il34 as a regulator of microglia development

**Fig. 3.**
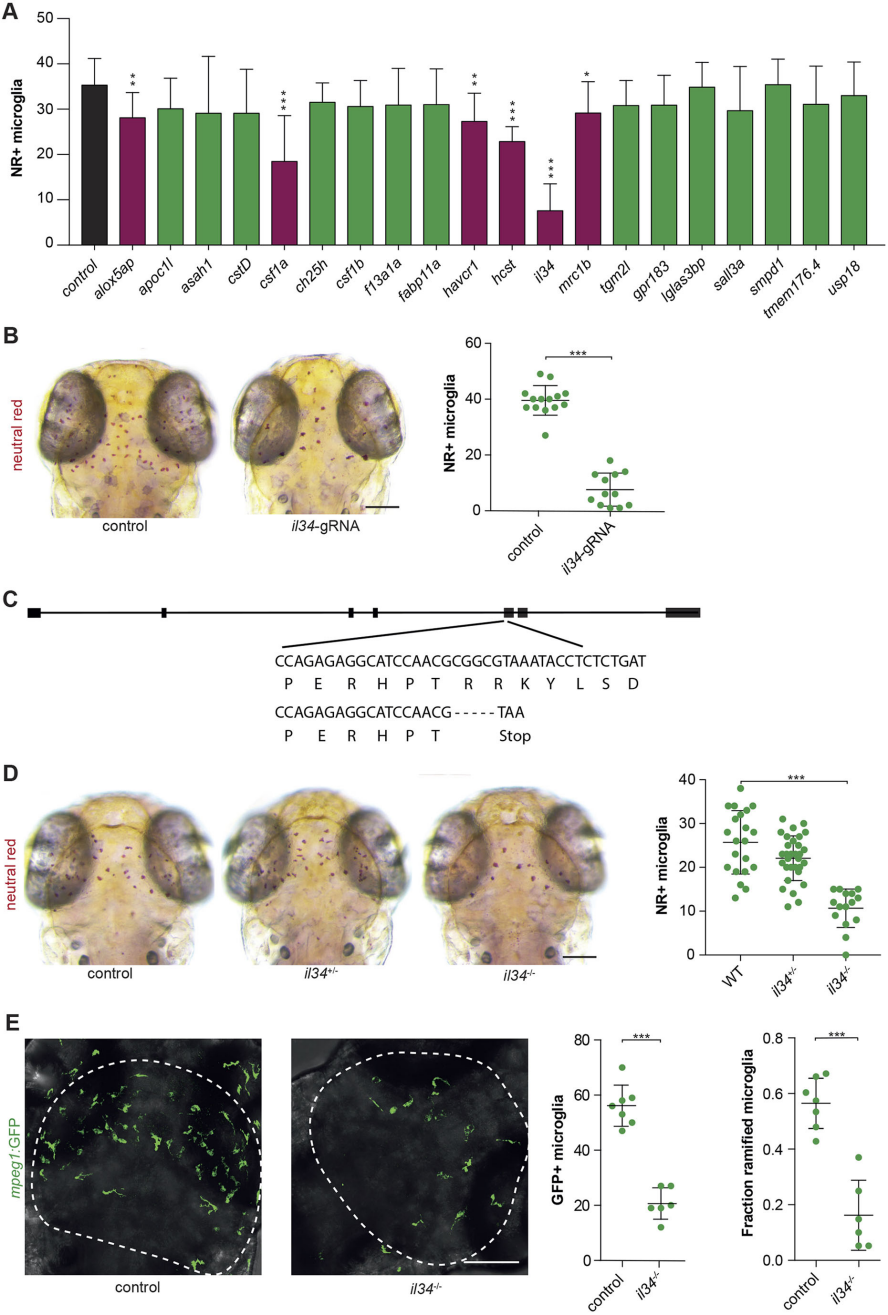
**Reverse genetic screen reveals zebrafish *il34* as a regulator of microglia development.** (A) Accumulated data from all gRNA injections showing the number of NR^+^ microglia as quantified with SpotNGlia. Magenta bars represent genes showing a significant reduction in microglia numbers upon CRISPR/Cas9-based targeting (black bar, control; green bars, genes with non-significant reduction in microglia numbers). (B) NR^+^ microglia numbers in 3 dpf zebrafish larvae injected with gRNA-Cas9 RNPs targeting *il34*. Controls in A and B are non-injected WT larvae. (C) A −5 bp deletion in exon 1 of *il34* directly introduces a stop codon. (D) NR^+^ microglia numbers in *il34* mutants with a premature stop codon in exon 5 and their heterozygous and WT siblings at 3 dpf. (E) GFP^+^ microglia in the optic tecti (dashed lines) of 3 dpf *il34* mutants and controls, and quantification of their numbers and the fraction of microglia containing more than one protrusion (ramified microglia). Controls in D and E are WT (*il34^+/+^*) larvae. *P<0.05, **P<0.01, ***P<0.001. One-way ANOVA and Student's *t-*test. Bonferroni correction for multiple testing. Scale bars: 100 µm. Each dot represents one larva. Error bars represent s.d.

To identify new microglia regulators using direct CRISPR/Cas9 targeting and microglia phenotyping by SpotNGlia, we targeted 20 candidate genes individually. These genes were selected based on either our recently identified zebrafish microglia transcriptome (e.g. *slco2b1*, *hcst* and *mrc1b*), microglia-expressed genes with a connection to brain disease (e.g. *usp18*), or genes that can affect microglia in a non-cell-autonomous manner (Csf1r-ligand-encoding genes *il34*, *csf1a* and *csf1b*) (Fig. 3A; Fig. S1, Table S1) (Oosterhof et al., 2017). Next, gRNAs were designed to effectively target these genes in one of their first exons. Cas9-gRNA RNPs targeting candidate genes were injected in fertilized oocytes, after which they were NR stained at 3 dpf, phenotyped and genotyped by Sanger sequencing, followed by indel decomposition using TIDE (Table S1) (Brinkman et al., 2014). We did not observe obvious signs of developmental delay, morphological abnormalities or increased mortality upon Cas9-gRNA RNP injections, indicating that the observed microglia phenotypes were not due to Cas9-gRNA toxicity. The gRNAs for six of the targeted genes caused a significant reduction in the number of NR^+^ microglia (Fig. 3A). The largest decrease in NR^+^ microglia numbers was observed in embryos in which the zebrafish homolog of IL34 was targeted (Fig. 3A,B) (Wang et al., 2013).

To validate our approach and confirm that this microglia phenotype is caused by loss of *il34* function, we generated a premature stop codon in exon 5 of the *il34* gene (Fig. 2C). NR labeling of homozygous *il34* mutants at 3 dpf revealed a ∼60% reduction in NR^+^ microglia compared with wild-type siblings, suggesting that this is a loss-of-function allele (Fig. 3D). Similarly, live imaging of GFP^+^ microglia, driven by the *mpeg1* (also known as *mpeg1.1*) promoter, in the optic tecti of *il34* mutants showed lowered microglia numbers compared with controls (Fig. 3E). In mice, *Il34* knockout led to slightly different outcomes, causing, in one study, lowered microglia numbers in early postnatal development that remained low into adulthood and, in another study, only reduced adult microglia numbers (Greter et al., 2012; Wang et al., 2012). Therefore, the precise role of IL34 in early microglia development remains ambiguous. In addition, the precise role of IL34 in adult microglia has not been described yet (Greter et al., 2012; Wang et al., 2012). Our results are consistent with an evolutionarily conserved role for *Il34* in early microglia development (Wang et al., 2012). This is further supported by a concurrent study in which, using another premature stop mutation in *il34*, the authors showed a similar reduction in microglia numbers at the same developmental stage (Wu et al., 2018). Interestingly, the receptor for Il34, Csf1r, has two other ligands in zebrafish: Csf1a and Csf1b. To determine whether the other Csf1r ligands also affect early microglia development, we generated stable frameshift mutants for *csf1a* and *csf1b* (Fig. S3). However, individual *csf1a* and *csf1b* mutants did not show reduced microglia numbers (Fig. S2A,B) (Wu et al., 2018). Surprisingly, larvae containing mutations in both zebrafish *csf1* homologs, *csf1a* and *csf1b* (*csf1a^−/−^b^−/−^*), also showed no reduction in microglia numbers (Fig. S2C). As the mutants presented with the absence of yellow pigment cells, known as xanthophores, a phenotype also observed in *csf1ra^−/−^* mutants, this suggests that the *csf1a^−/−^b^−/−^* fish are loss-of-function mutants (Parichy et al., 2000; Parichy and Turner, 2003; Patterson et al., 2014; Patterson and Parichy, 2013). Many *in vitro* studies have shown that CSF1 can induce proliferation of myeloid cells (Stanley and Chitu, 2014; Tushinski and Stanley, 1985). Consistently, we found that overexpression of Csf1a [*Tg(hsp70l:csf1a-IRES-nlsCFP)* (Patterson and Parichy, 2013)] caused an increase in microglia numbers quantified (Fig. S2D). These data suggest that increased Csf1a is capable of influencing microglia numbers, but Csf1 is not essential for early microglia development. In all, the loss of Il34, but not Csf1, causes a reduction in microglia numbers in 3 dpf zebrafish.

### Il34 facilitates the distribution of macrophages, without affecting their proliferation

**Fig. 4.**
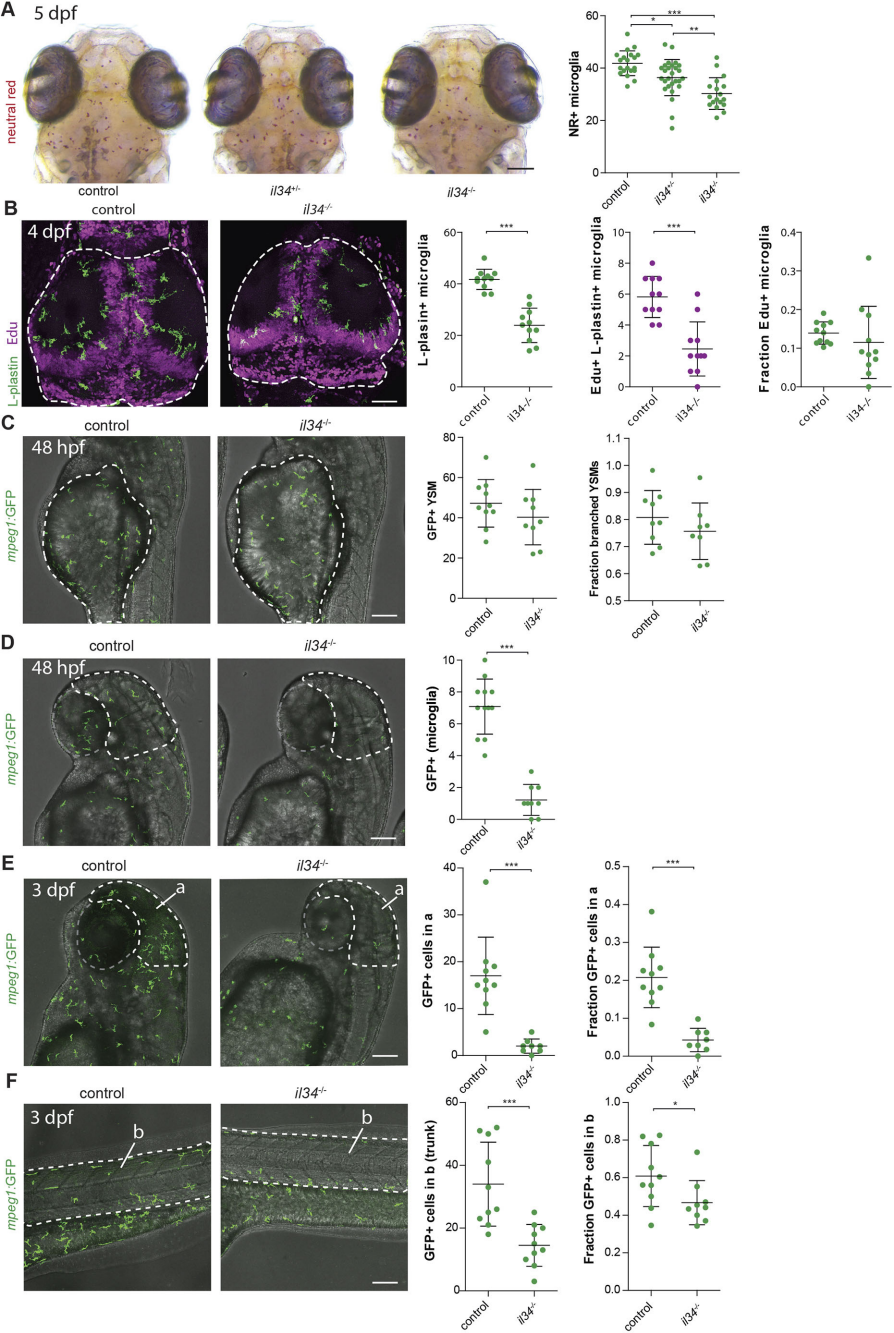
**Il34 does not affect proliferation but does affect** **the distribution of YSMs to target organs.** (A) NR^+^ microglia numbers in *il34* mutants and their heterozygous and WT siblings at 5 dpf. (B) EdU/L-plastin staining of microglia in the optic tecti (dashed lines) of 4 dpf *il34* mutants and WT controls, and quantification of microglia numbers, EdU^+^ microglia numbers and the fraction of EdU^+^ microglia among total numbers. (C) *In vivo* imaging of GFP^+^ macrophages located on the yolk sac (dashed lines) in *il34* mutants and WT controls, transgenic for *mpeg1-GFP*, and quantification at 48 hpf. YSMs with more than one protrusion were counted as branched YSMs. (D) *In vivo* imaging of *mpeg1*-GFP^+^ macrophages located in the head region (dashed lines) in *il34* mutants and WT controls, and quantification at 48 hpf. (E) *In vivo* imaging of GFP^+^ macrophages located in the head region (dashed lines) in *il34* mutants and WT controls, and quantification at 3 dpf. a, outline of the head region. (F) *In vivo* imaging of *mpeg1*-GFP^+^ macrophages located in the tail (dashed lines) in *il34* mutants and WT controls, and quantification. b, outline of the embryonic region/trunk region. Scale bars: 100 µm. *P<0.05, **P<0.01, ***P<0.001. One-way ANOVA and Student's *t-*test. Each dot represents one larva. Error bars represent s.d.

**Fig. 5.**
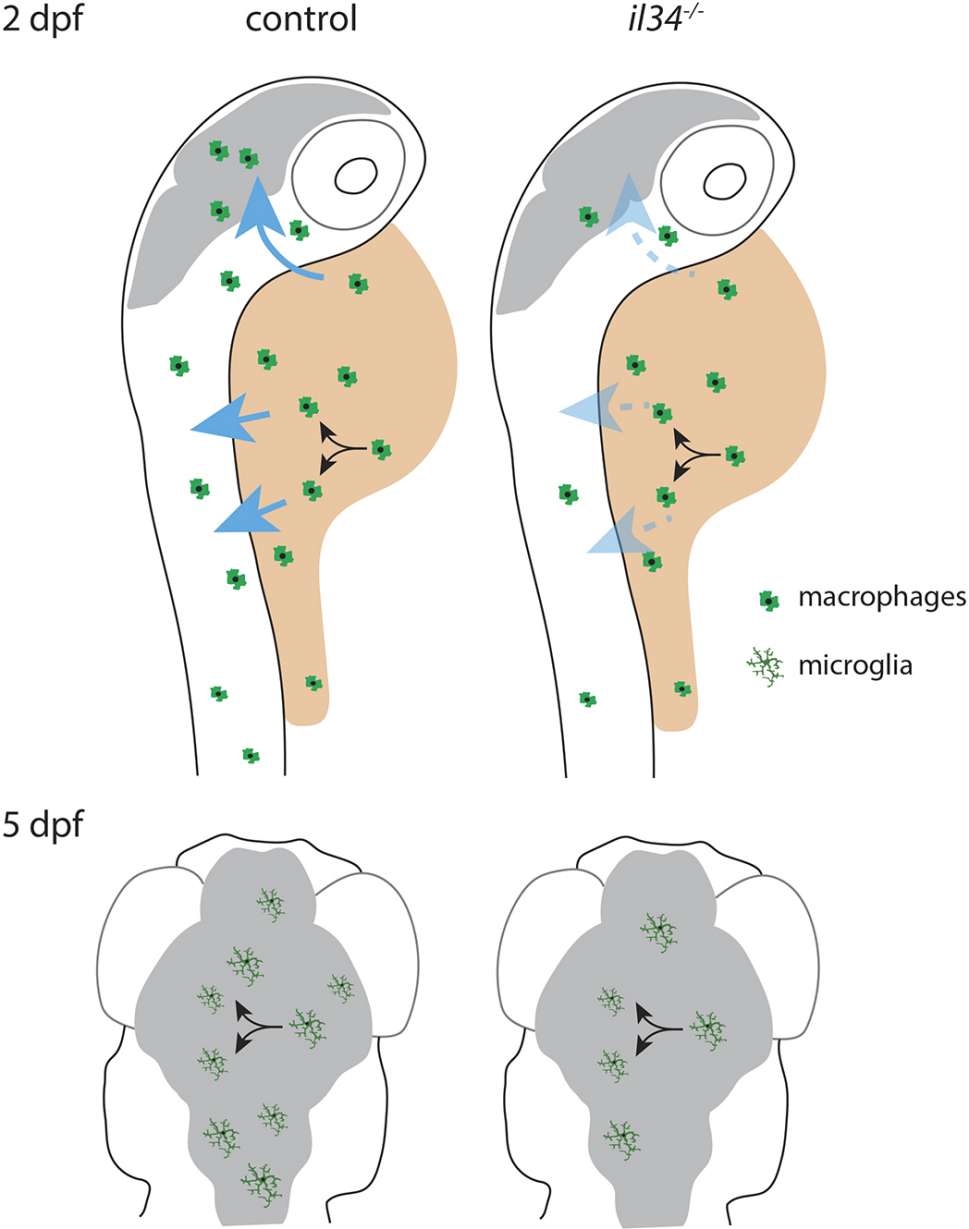
**Schematic representation of the role of Il34 in distribution of YSMs to target organs.** (Upper panels) In control 2-day-old larvae, macrophages from the yolk migrate into the embryonic tissue guided by Il34 (blue arrows). In *il34* mutants, yolk sac macrophages largely fail to migrate towards the embryonic tissues, leading to reduced colonization of, in particular, the brain. (Lower panels) Microglia are less abundant in 5-day-old larvae due to *il34* deficiency, but continue to proliferate independently of Il34. Blue arrows indicate the direction of macrophage migration into the embryo, induced by Il34. Black arrows indicate cell division. Gray indicates the area of the brain.

In mice, tissue-resident macrophages of the skin, known as LCs, are highly dependent on IL34/CSF1R signaling for their maintenance and self-renewal (Greter et al., 2012; Wang et al., 2012, 2016)*.* We therefore hypothesized that Il34 in zebrafish might regulate the proliferative expansion of microglia, similar to LCs in mice, leading to the lower microglia numbers we observed. Microglia numbers increase sharply after 3 dpf and, to determine whether microglia numbers remained lower over time, we quantified NR^+^ microglia also at 5 dpf (Fig. 4A). Surprisingly, compared with 3 dpf, microglia numbers in *il34*^−/−^ mutants were closer to those of controls at 5 dpf (∼30% reduction at 5 dpf versus ∼60% reduction at 3 dpf). To determine whether the increase in numbers was due to the continuation of seeding the brain or proliferative expansion, we performed 5-ethynyl-2'-deoxyuridine (EdU) pulse labeling between 3 dpf and 4 dpf. EdU/L-plastin double labeling showed reduced microglia and reduced Edu^+^ microglia, but the fraction of EdU^+^ microglia did not differ between *il34* mutants and controls (Fig. 4B; Fig. S4A). Thus, loss of *il34* does not change the proliferative fraction of microglia; therefore, the decreased microglia numbers are unlikely explained by a defect in proliferation. Since the decrease in microglia numbers in *il34* mutants compared with controls was largest at 3 dpf, Il34 likely affects YSMs, including microglia progenitors, preceding brain colonization. Indeed, Wu and colleagues show that Il34 deficiency causes impaired colonization by failing to attract YSMs to enter the brain in a Csf1ra-dependent mechanism (Wu et al., 2018). We used live imaging to visualize *mpeg1*-GFP^+^ YSMs, which are the progenitors of microglia but also of many other macrophages at this stage. At 2 dpf, YSM numbers and morphology were not different between *il34* mutants and controls (Fig. 4C). Thus, reduced microglia numbers are likely not attributed to reduced YSM numbers. Therefore, impaired migration of *il34*-deficient YSMs towards the brain could explain the lower microglia numbers. Imaging in the rostral/head region at 2 dpf showed an >80% decrease in the number of macrophages/microglia, suggesting that *il34* is indeed involved in the recruitment of YSMs to the brain (Fig. 4D). To determine whether this effect is exclusive to microglia, we determined the fraction of total macrophages that was found in the head or in the trunk region at 3 dpf. This showed again an ∼80% reduced infiltration of microglia in the brain in *il34* mutants compared with controls. Colonization of the trunk was also decreased in *il34* mutants compared with controls, but to a lesser extent (∼ 25% reduction) (Fig. 4E,F; Movie 1). This was confirmed by time-lapse imaging of *il34* mutants and controls, which showed largely reduced colonization of all embryonic regions (Movie 1). In addition, we observed frequent proliferative events between 2 and 3 dpf, both in control but also in *il34* mutant larvae, and therefore proliferation of *il34^−/−^* YSMs caused an increase in the number of YSMs that had infiltrated the tissue (Movie 1). Analysis of entire larvae at 8 dpf revealed that total macrophage numbers were not reduced in *il34* mutants, suggesting normal macrophage development and expansion. However, whereas in control fish almost 60% of the total macrophages were found to have migrated away from the hematopoietic sites into the embryonic tissues, in *il34* mutant fish only 40% of the macrophages colonized the embryo. Therefore, loss of Il34 affects the distribution of macrophages into various embryonic tissues including the brain, analogous to the effect of IL34 on the maintenance and development of LCs, as described in mice (Greter et al., 2012; Wang et al., 2012, 2016) (Fig. 5).

## DISCUSSION

In this study, we developed a scalable CRISPR/Cas9-based reverse genetic screening pipeline using semi-automated image quantification to identify new regulators of microglia biology using zebrafish embryos. We showed that direct genetic targeting of known microglia regulators, including *csf1ra* and *spi1b*, by Cas9/gRNA injections in zebrafish embryos phenocopies previously identified microglia mutants. We next developed a software tool (SpotNGlia) that allows for automated phenotyping by quantification of NR^+^ microglia. As zebrafish are well suited for *in vivo* drug discovery, our strategy could potentially also be used to identify small molecules affecting microglia development (Zon and Peterson, 2010). Using this pipeline, we here tested 20 candidate genes for a role in microglia development and found six genes significantly affecting microglia numbers when mutated. Loss of *il34* function caused the largest decrease in microglia numbers, which we confirmed by analysis of stable *il34* mutants. Furthermore, we uncovered Il34 as a regulator of distribution of tissue macrophages, needed to recruit YSMs to the brain and other embryonic tissues.

Even though we here examined 20 genes, there are several ways to increase the throughput of our screening strategy. First, mounting of the injected zebrafish larvae and subsequent image acquisition are the most time-consuming parts of our pipeline. NR-stained larvae were manually embedded in low-melting-point agarose before imaging, which restricts the number of animals that can be screened per day. Automated imaging systems that can load zebrafish larvae from liquid medium in multi-well plates and image them in the orientation of interest in glass capillaries could overcome this hurdle (Pardo-Martin et al., 2010). Together with the SpotNGlia tool, this would permit a significantly increased screening throughput and efficiency. Additionally, we aimed to achieve maximal CRISPR/Cas9 mutagenic efficiency for individual genes of interest, and therefore targeted individual genes. Shah et al. previously reported a strategy by which pools of up to eight gRNAs are injected simultaneously to target multiple genes at once (Shah et al., 2016), which could lead to reduced targeting efficiency of the individual gRNAs. Although a pooling strategy could significantly increase the number of genes that can be screened, we observed that, especially for genes with a relatively subtle microglia phenotype, a high mutagenic efficiency increases the chance of detecting the phenotype. Additionally, due to the clonal nature of hematopoietic progenitors, including yolk sac macrophages, a high targeting efficiency is likely required, because non-targeted cells could expand and compensate for mutated cells.

IL34 is one of two ligands of CSF1R, a main regulator of development of the macrophage lineage (Stanley and Chitu, 2014). Even though adult *Il34*-deficient mice have fewer microglia, and no LCs, the precise role of IL34 in microglia development is unclear. Wang and colleagues showed that neonatal *Il34*^−/−^ mice have lower microglia numbers, whereas Greter et al. showed normal microglia numbers in *Il34*^−/−^ mice throughout embryonic development (Greter et al., 2012; Wang et al., 2012). The exact function of IL34 in microglia development *in vivo*, and how this may differ from that of CSF1, therefore, remains ambiguous. These discrepancies could be attributed to factors such as genetic background, or slightly different methods leading to different interpretations regarding the role of IL34 in embryonic and early postnatal microglia numbers (Greter et al., 2012; Wang et al., 2012).

Our data revealed a ∼60% reduction in microglia numbers in *il34* mutant larvae at 3 dpf, indicating that *il34* is required for early microglia development in zebrafish. We show that, upon arrival in the brain, between 3 dpf and 5 dpf, microglia numbers increase by proliferation in both controls and *il34* mutants, suggesting that the proliferative capacity of microglia is not affected by the loss of *il34* (Fig. 5). In addition, YSM numbers were not affected by *il34* deficiency, indicating that there is a defect in the colonization of the embryonic brain, likely due to a failure to attract YSMs expressing Csf1ra and/or Csf1rb. Consistent with this, analysis of migration towards the brain at both 2 dpf and 3 dpf showed that much fewer microglia colonized the brains of *il34*-deficient larvae. Our findings are consistent with a concurrent paper, in which the authors show that nervous system expression of Il34 can attract YSMs to migrate into the brain by the Il34/Csf1 receptor Csf1ra (Wu et al., 2018). However, we additionally found that distribution of *il34* mutant YSMs into trunk regions was reduced, indicating that the effect of Il34 is not limited to microglia, but also affects the migration and colonization of other tissue-resident macrophages. Consistent with this idea, recent single-cell RNA sequencing studies show widespread expression of *il34* mRNA in early embryonic zebrafish (Wagner et al., 2018). At 24 hours post-fertilization (hpf), *il34* is already expressed in e.g. the brain, but also in muscle, heart, pharyngeal arches, epidermis and neural crest (Wagner et al., 2018). In mouse, IL34 is also expressed in the brain during embryonic development (E11.5, and possibly earlier) and, for example, in the epidermis (Greter et al., 2012; Wei et al., 2010). This early expression in brain and other cells supports our model that Il34 attracts YSMs towards the brain and into other parts of the embryo, including the epidermis.

We previously showed that mutants for both receptors, Csf1ra and Csf1rb, lack all microglia, in contrast to Csf1ra mutants, which have fewer microglia only in early development. Therefore, the expansion of microglia following colonization of the brain is likely regulated by other, possibly compensatory or redundant, factors, including through the CSF1 homologs Csf1a or Csf1b (Dai et al., 2002; Oosterhof et al., 2018). Although we repeatedly identified a decrease in microglia numbers in *csf1a* gRNA-injected zebrafish, we did not identify a change in *csf1a* mutants, generated with the same gRNA. Our data are consistent with an already-published *csf1a* mutant line that also shows normal microglia numbers (Wu et al., 2018). Even when we combined *csf1a* and *csf1b* frameshift mutations, we did not find reduced microglia numbers. The pigmentation phenotype observed in *csf1a^−/−^b^−/−^* larvae, and not in the individual mutants, suggests that the mutations in *csf1a* and *csf1b* are loss of function and possibly compensate for each other. This suggests that genetic compensation, where alternative pathways are upregulated upon mutation of exonic regions, does not occur regarding the pigment phenotype (El-Brolosy et al., 2018; Rossi et al., 2015a). It is likely that loss of all three ligands (Csf1a, Csf1b and Il34) leads to a similar microglia phenotype, as observed in *csf1r* knockouts. Our *csf1* gRNA injections reduce microglia numbers, and overexpression of *csf1a* increases microglia numbers. Therefore, *csf1* in zebrafish seems capable of influencing microglia numbers. We cannot currently explain the discrepancy between results obtained with gRNA injections and stable mutants, and it is possible that genetic compensation for *csf1*, perhaps by other ligands, could occur in *csf1* mutants regarding microglia. This stresses the importance of using multiple independent approaches to detect false-positive, and also false-negative, results (El-Brolosy et al., 2018; Kok et al., 2015; Rossi et al., 2015a).

*Csf1* and *Il34* were both found to be expressed in the adult mouse brain, although in non-overlapping regions; however, during early embryonic development, IL34 expression precedes CSF1 expression in mice (Nandi et al., 2012; Wang et al., 2012)*.* This corroborates our findings that Il34 acts as a beacon for YSMs to migrate towards the brain, whereas loss of Csf1 appears not to affect microglia numbers at this early developmental stage. In *il34* mutants, YSMs that arrive in the brain at 3 dpf start to proliferate and reach 70% of control levels at 5 dpf. Time-lapse imaging showed frequent proliferative events in other tissues of *il34* mutants as well. Thus, we find that, whereas Csf1 appears able to influence microglia numbers, it seems not essential for early embryonic microglia development. On the other hand, *il34* is a critical, non-cell-autonomous regulator of seeding of the brain and other organs by YSMs, but does not appear to be required for their proliferation.

In conclusion, we here present a scalable reverse genetic screening method for the identification of novel regulators of microglia development and function. Microglia are key players in brain disease and there is strong evidence that microglia defects can be a primary cause of brain disease (Oosterhof et al., 2018; Paloneva et al., 2002; Rademakers et al., 2011; Sundal et al., 2012). Replenishing microglia; for example, by hematopoietic stem cell transplantation, can provide therapeutic benefit in human brain diseases. Better understanding of microglia development, and acquisition of their specific cell fate *in vivo*, could lead to improved strategies to replace defective microglia. However, the mechanisms and genes regulating microglia development and function are still largely unknown. Therefore, better understanding of microglial gene functions could be a valuable step in the elucidation of mechanisms underlying microglial biology. As zebrafish larvae have proven their suitability for drug discovery, SpotNGlia automated analysis software in combination with automated imaging systems could also be used to screen for compounds affecting microglia (MacRae and Peterson, 2015). In all, we identified *il34* as a regulator of tissue-resident macrophage distribution, primarily affecting macrophage colonization of the zebrafish embryonic brain by affecting the recruitment of YSMs to target organs including the brain. Our reverse genetic screening pipeline can be used to address genetic regulation of microglia development and function, and identify regulators essential to generate functional microglia *in vivo*.

## MATERIALS AND METHODS

### Fish care

For all experiments, Tg(*mpeg1*:*EGFP*) fish expressing GFP under the control of the *mpeg1* promotor or Tg(*Neuro*-*GAL4*, *UAS*:*nsfB*-*mCherry*, *mpeg1*:*EGFP*) with neuronal specific nitroreductase expression, transgenic zebrafish lines were used (Ellett et al., 2011). Zebrafish embryos were kept at 28°C on a 14 h/10 h light-dark cycle in HEPES-buffered E3 medium. At 24 hpf, 0.003% 1-phenyl 2-thiourea (PTU) was added to prevent pigmentation. For overexpression of Csf1a, we used *Tg(hsp70l:csf1a-IRES-nlsCFP)^wp.r.t4^* fish kindly provided by David Parichy (University of Virginia, Charlottesville, VA, USA) (Patterson and Parichy, 2013). Fish were heat shocked twice at 37°C for 1 h at 4 dpf. After heat-shock treatment, fish were selected on CFP expression and divided into CFP^–^ controls and CFP^+^ fish. In this paper, we describe three new mutant fish lines: *il34^re03/re03^* containing a 5 bp deletion in exon 5 (Fig. 2C); *csf1a^re05/re05^* containing a 4 bp insertion in exon 2; *csf1b^re06/re06^* containing a 4 bp deletion in exon 2, and a version in the *csf1a^re05/re05^* background containing a −3 bp deletion and +28 insertion, leading to a +25 bp insertion causing a frameshift in exon 2 (*csf1b^re07/re07^*) (Fig. S4).

### Ethics statement

Animal experiments were approved by the Animal Experimentation Committee at Erasmus University Medical Center, Rotterdam, The Netherlands. Zebrafish embryos and larvae were anesthetized using tricaine and euthanized by ice water.

### sgRNA synthesis

To design single-guide RNAs (sgRNAs), the online program CRISPRscan (www.crisprscan.org) was used (Moreno-Mateos et al., 2015). The gRNAs were designed to target exons, except for exon 1, to be as close as possible to the transcription start site and to have no predicted off-target effects. The sgRNAs were generated from annealed primers, one containing a minimal T7 RNA polymerase promoter, the target sequence, and a tail-primer target sequence and a generic tail-end primer (Vejnar et al., 2016). To generate primer dimers, the FastStart™ High Fidelity PCR System from Sigma-Aldrich was used. A solution was prepared, containing 1 mM forward sgRNA oligonucleotide, 1 mM reverse oligonucleotide consisting of 20-nucleotide overlap with sgRNA oligonucleotide and the Cas9-binding part, 0.8 mM dNTPS, 1× FastStart Buffer and 6.25 U/µl FastStart Taq polymerase in 20 µl total volume. Annealed DNA oligonucleotide dimers were generated by denaturation at 95°C for 5 min followed by annealing by reducing the temperature by 1°C per second over 20 s to 75°C and extension at 72°C for 10 min. The gRNAs were synthetized from annealed DNA oligonucleotides, containing a minimal T7 RNA polymerase promoter, with the mMESSAGE mMACHINE™ T7 ULTRA Transcription Kit (Invitrogen), according to the manufacturer's instructions.

### Cas9/gRNA complex injections into zebrafish larvae

The SP-Cas9 plasmid used for the production of Cas9 protein was Addgene plasmid #62731, deposited by Niels Geijsen (D'Astolfo et al., 2015). Cas9 nuclease was synthetized as described (D'Astolfo et al., 2015). gRNA (600-900 ng) was mixed with 4 ng Cas9 protein to form active gRNA-Cas9 RNPs. Next, 0.4 µl of 0.5% Phenol Red (Sigma-Aldrich) was added, and the volume was adjusted with 300 mM KCl to a total volume of 6 µl. Approximately 1 nl of the mix was injected into fertilized zebrafish oocytes. For the creation of mutant lines, CRISPants were grown to adulthood and outcrossed to the AB background, and Sanger sequencing was used to identify mutations.

### NR staining and imaging

To label microglia, 3 dpf or 5 dpf larvae were incubated in E3 medium containing NR (Sigma-Aldrich) (2.5 µg/ml) for 2 h at 28°C, after which they were rinsed with E3 medium containing 0.003% PTU. Larvae were anesthetized with 0.016% MS-222 and embedded in 1.8% low-melting-point agarose in E3 medium with the dorsal side facing upwards. Serial images (3-6) in the *z*-plane were acquired with a Leica M165 FC microscope using the 12× dry objective and a Leica DFC550 camera.

### Larvae genotyping (Sanger sequencing)

#### Lysis

Zebrafish larvae were euthanized and placed in single tubes containing 100 µl lysis buffer (0.3% 1 M KCl, 1% 1 M Tris-HCl pH 9.0, 0.1% Triton, 0.15 mg/ml Proteinase K) per larva. The mix was incubated at 55°C for 10 min and 95°C for 10 min. The lysate was centrifuged for 5-10 min at 4000 rpm (1500 ***g***), and 1 µl was used for PCR.

#### Sanger sequencing to determine CRISPR/Cas9 targeting efficiency

For Sanger sequencing, 500 bp long PCR products were obtained. For the sequencing reaction, a BigDye^®^ Terminator v3.1 Cycle Sequencing Kit from Applied Biosystems was used. The product was placed on Sephadex^®^ columns (Sigma-Aldrich) and centrifuged at 910 rcf (910 ***g***) for 5 min. The ABI 3130 genetic analyzer from Applied Biosystems was used for Sanger sequencing. To assess the indel spectrum and frequencies at the target locus, we used the program TIDE developed by the Netherlands Cancer Institute (NKI) (Brinkman et al., 2014).

### SpotNGlia

The SpotNGlia software tool was developed in MATLAB (MathWorks, Natick, MA, USA). Its full source code and a technical description of how to use the tool is available from GitHub (https://github.com/samuelgeurts/SpotNGlia). The software is released under the GNU General Public License. A brief description of the three main steps (pre-processing, brain segmentation and microglia detection) performed by the software is given below.

#### Pre-processing

Images acquired from NR-labeled larvae (*n*=50) were used to optimize the algorithm. For each larva, 3-6 images were taken at different depths of focus. Color channels were realigned by finding the translation that maximizes the correlation coefficient (Evangelidis and Psarakis, 2008). To remove the background, the triangle thresholding method was used (Zack et al., 1977). Next, we generated an all-in-focus image with extended depth of field (Forster et al., 2004).

#### Brain segmentation

The orientation of the fish was determined by maximizing the correlation coefficient between the image and a mirrored version of itself, yielding the larva's rotation angle. The translation parameters were found by directly correlating the image to a template image, which was established by averaging multiple aligned fish. Because of its near-circular shape, the optic tectum was segmented by performing a polar transformation, after which the edges of the optic tectum were found by using Dijkstra's algorithm (Dijkstra, 1959; Zinser and Komitowski, 1983). The brain edge becomes an approximately straight line in polar coordinates if it is transformed with respect to the center of the optic tectum, which we obtained from the template image. To make it applicable for the shortest path algorithm, the image was correlated with a small image, similar to the average appearance of the brain edge in the polar image. Also, *a priori* information of the training set was used to exclude locations in which the brain edge cannot be. After Dijkstra's algorithm was applied, the found path was transformed back, resulting in the brain edge coordinates.

#### Microglia detection

To identify NR^+^ microglia, a multi-scale wavelets product was computed on the green channel of the image, which contains the highest contrast for the NR signal (Olivo-Marin, 2002). Multiple smoothed images from a single fish image were produced with increasing spatial scale. Subtracting adjacent smoothed images resulted in sub-band images containing different scales of detail present in the image. Sub-band images in the range of the microglia spot size were combined by pixel-wise multiplication to obtain an image with only high values at the location of the spots, i.e. the multi-scale wavelet product. A threshold on the multi-product image was applied to obtain a binary image to determine the spots. The identified spots were discriminated further on typical color and size obtained from the training set, resulting in accurate quantification of microglia numbers. All NR quantifications were performed using SpotNGlia, except for the 5 dpf larvae in Fig. 4A and Fig. S2D.

### Immunofluorescence staining

Immunohistochemistry was performed as described (van Ham et al., 2012, 2014). Briefly, larvae were fixed in 4% paraformaldehyde (PFA) at 4°C overnight. Subsequently, they were dehydrated in 100% MeOH, stored at −20°C for at least 12 h and rehydrated in PBS. Then, they were incubated in blocking buffer [10% goat serum, 1% Triton X-100 (Tx100), 1% bovine serum albumin (BSA), 0.1% Tween-20 in PBS] for 3 h at 4°C, before incubation in primary antibody buffer at 4°C overnight. Larvae were washed in 10% goat serum, 1% Tx100 in PBS and PBS containing 1% Tx100 for a few hours, followed by incubation in secondary antibody buffer at 4°C overnight. Primary antibody buffer contained 1% goat serum, 0.8% Tx100, 1% BSA and 0.1% Tween-20 in PBS. Secondary antibody buffer contained 0.8% goat serum, 1% BSA and PBS containing Hoechst. Primary antibody against L-plastin (1:500) was a gift from Yi Feng (University of Edinburgh, Edinburgh, UK). Secondary antibody was DyLight Alexa Fluor 488 (1:250).

### EdU pulse-chase protocol

Larvae of 3 dpf were placed in a 12-well plate in HEPES-buffered (pH 7.3) E3 medium containing 0.003% PTU and 0.5 mM EdU for 24 h. Next, larvae were fixed in 4% PFA at 4°C overnight, dehydrated in 100% MeOH and stored at −20°C for at least 12 h. They were then rehydrated in PBS in series and incubated in proteinase K (10 µg/ml in PBS) for 1 h at room temperature, followed by 15 min post-fixation in 4% PFA. Larvae were incubated in 1% dimethyl sulfoxide in PBS containing 0.4% Triton X-100 for 20 min. Then, 50 µl Click-iT™ (Invitrogen) reaction cocktail was added for 3 h at room temperature, protected from light. Samples were subjected to immunolabeling using L-plastin antibody (see ‘Immunofluorescence staining’ section).

### Confocal imaging

Intravital imaging was largely performed as previously described (van Ham et al., 2014). Briefly, zebrafish larvae were mounted as described for NR staining. The imaging dish containing the embedded larva was filled with HEPES-buffered E3 medium containing 0.016% MS-222. Confocal imaging was performed using a Leica SP5 intravital imaging setup with a 20×/1.0 NA water-dipping lens. Imaging of GFP and L-plastin labeled with Alexa Fluor 488 was performed using the 488 nm laser; imaging of EdU labeled with Alexa Fluor 647 was performed using the 633 nm laser. Analysis of imaging data was performed using ImageJ (Fiji) and LAS AF software (Leica). The sequence in which larvae were imaged (live imaging) was randomized to avoid any adverse effects due to the anesthetics or to mounting.

### Statistical analysis

For image processing and quantitative analysis, SpotNGlia, ImageJ and Prism (GraphPad) were used. Statistical significance was calculated using one-way ANOVA with Bonferroni correction or Student's *t*-tests. Error bars represent s.d. and *P*<0.05 was considered significant. Fish showing signs of developmental delay, improper staining or incorrect mounting and/or annotation by SpotNGlia were excluded.

## Supplementary Material

Supplementary information
