# Supplementary material for: Reverse genetic screen reveals that Il34 facilitates yolk sac macrophage distribution and seeding of the brain
Source: Dis Model Mech. 2019 Mar 8;12(3):dmm037762. doi: 10.1242/dmm.037762 (PMC6451432; doi:10.1242/dmm.037762)
Supplement: Supplementary information [file dmm-12-037762-s1.pdf]

SUPPORTING INFORMATION

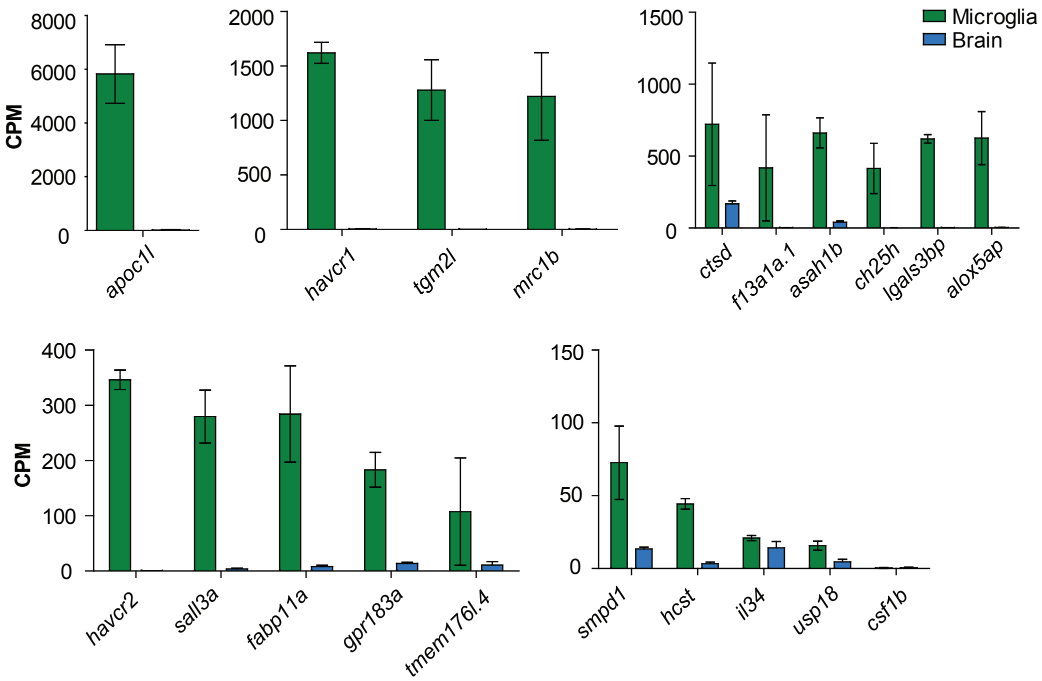

Fig S1. Expression of putative regulators of microglia development in the zebrafish brain.

Bar graphs represent expression values of putative microglia regulators in microglia (green) and other brain cells (blue) observed in the microglia transcriptome (27). Error bars represent s.d.

Disease Models & Mechanisms • Supplementary information

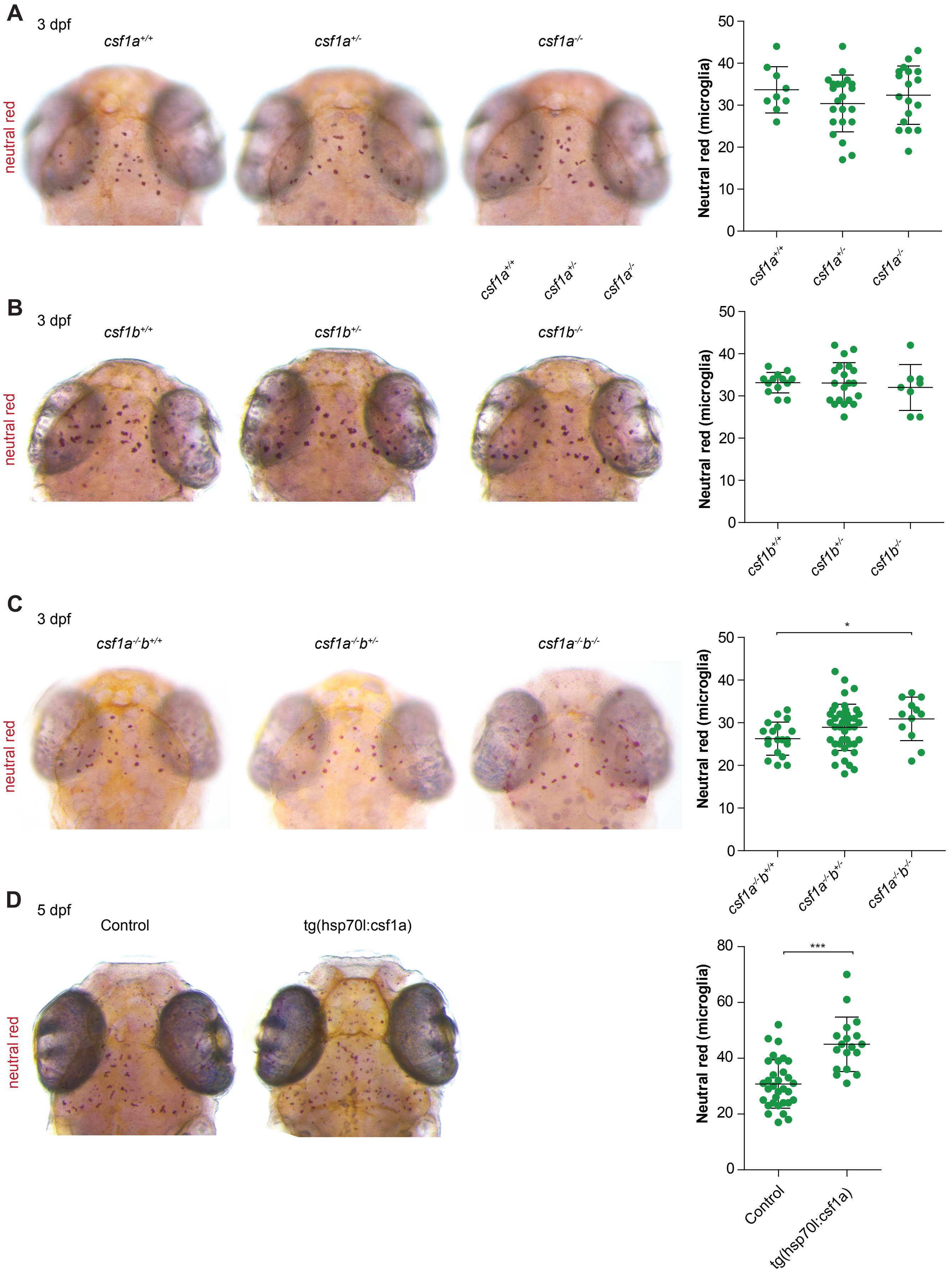
A
3 dpf
csf1a$^{+/+}$
csf1a$^{+/-}$
csf1a$^{-/-}$
neutral red
Neutral red (microglia)
B
3 dpf
csf1b$^{+/+}$
csf1b$^{+/-}$
csf1b$^{-/-}$
C
3 dpf
csf1a$^{-/-}$b$^{+/+}$
csf1a$^{-/-}$b$^{+/-}$
csf1a$^{-/-}$b$^{-/-}$
*
D
5 dpf
Control
tg(hsp70l:csf1a)
***

Fig S2. Mutations in *csf1* alleles do not affect microglia numbers at 3 dpf

(A) *csf1a* heterozygous cross shows similar numbers of microglia in *csf1a*$^{wt/wt}$, *csf1a*$^{wt/+4}$, and *csf1a*$^{+4/+4}$ larvae at 3 dpf. (B) *csf1b* heterozygous cross shows similar numbers of microglia in *csf1b*$^{wt/wt}$, *csf1b*$^{wt/-4}$, and *csf1b*$^{-4/-4}$larvae at 3 dpf. (C) Loss of *csf1b* in the *csf1a*$^{+4/+4}$ background does not change the numbers of microglia at 3 dpf. *csf1a*$^{+4/+4}$ and *csf1a*$^{+4/+4}$*csf1b*$^{+25/+25}$ show similar numbers of microglia. (D) Overexpression of Csf1a increases microglia numbers at 5 dpf. * $p < 0.05$, *** $p < 0.001$. One-way anova and *t*-test. Each dot represents one larvae. Error bars represent S.D.

Disease Models & Mechanisms • Supplementary information

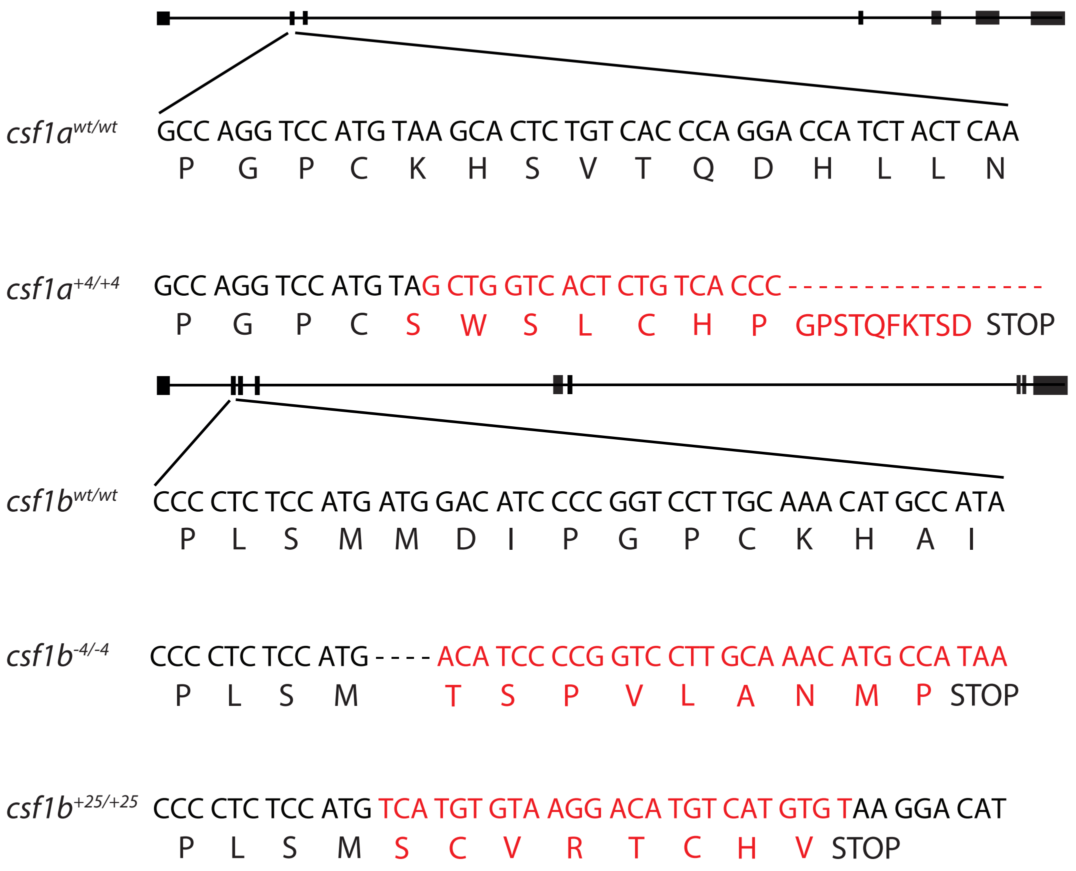

Fig S3. Schematic representation of out of frame mutations in *csf1a* and *csf1b*

Disease Models & Mechanisms • Supplementary information

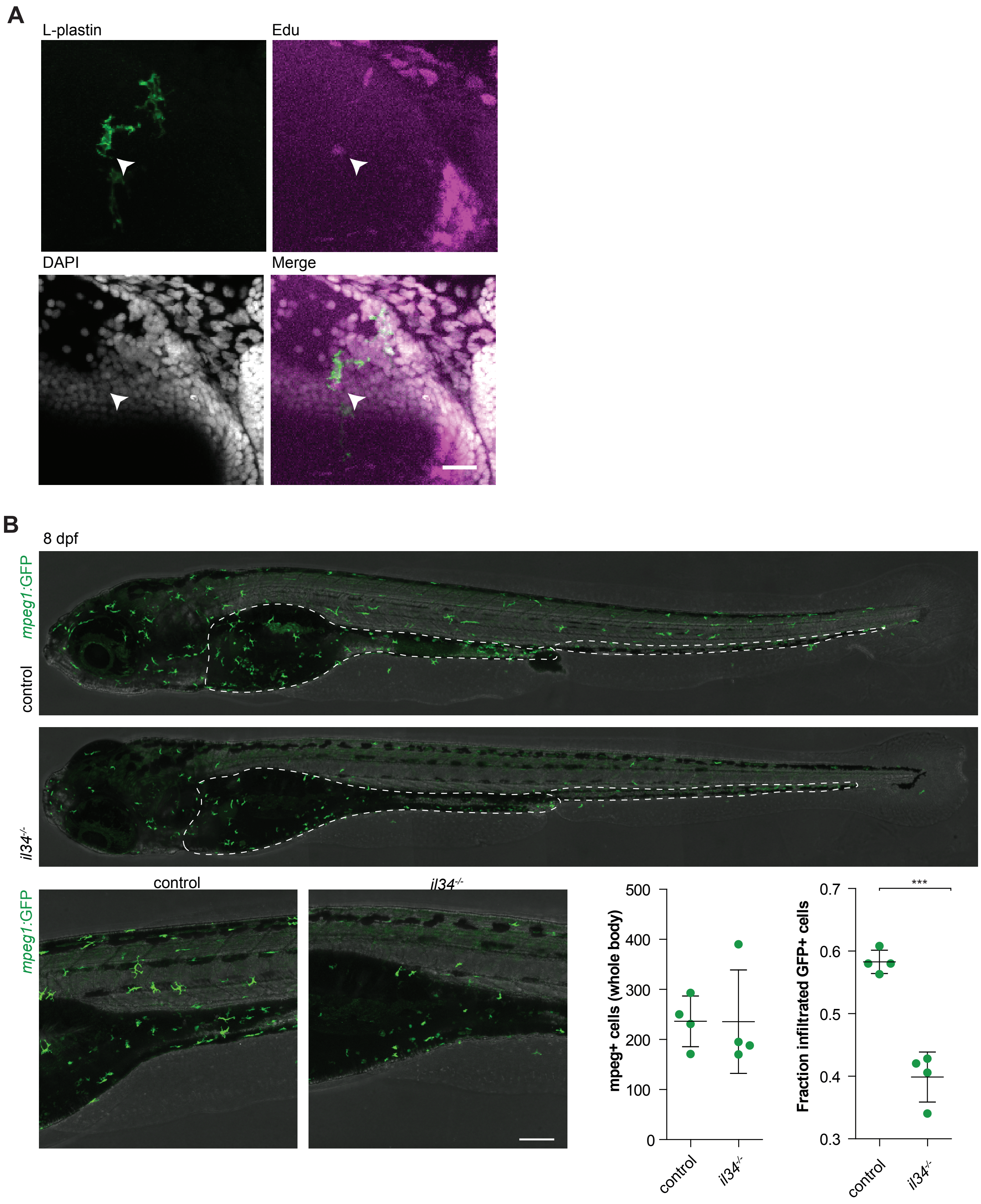
A
L-plastin
Edu
DAPI
Merge
B
8 dpf
mpeg1:GFP
control
il34-/-
control
il34-/-
mpeg1:GFP
mpeg+ cells (whole body)
500
400
300
200
100
0
control
il34-/-
Fraction infiltrated GFP+ cells
0.7
0.6
0.5
0.4
0.3
***
control
il34-/-

Fig S4. Loss of Il34 reduces overall tissue colonization by macrophage progenitors

(A) Representative images of an individual Lplastin+/Edu+ microglia related to Fig. 4B. Arrowheads depict the Lplastin+/Edu+ microglia. Scale bar represents 20 µm (B) Representative images of 8 dpf wildtype control and *il34* mutant fish with in addition a magnified image of the trunk region. Quantification of total macrophage numbers, and the fraction that infiltrated the embryo (number of macrophages counted outside the area depicted by the dashed line divided by total macrophage count). Scale bar represents 100 µm, *** $p < 0.001$, *t*-test. Each dot represents one larvae. Error bars represent s.d.

Table S1. gRNAs and their mutagenic efficiencies.

Gene	gRNA sequence	Efficiency	R^2
alox5ap	GGATACGTACCCTACATTTC	32%	0.98
apoc1l	GGCCCAGGAGGAGCCCACAC	84%	0.90
asah1	AGCTGGAGGATTGCAGAAGT	52%	0.92
cstD	CGCGTCGGACGTGCAGAAAA	74%	0.90
csf1a	TGGGTGACAGAGTGCTTACA	91%	0.91
ch25h	GGTAGACTGTAATTGAGAAG	64%	0.89
csf1b	AGGACCGGGGATGTCCATCA	79%	0.79
f13a1a	GGTCAAACAAGATGTCGATG	30%	0.96
fabp11a	GGAGTCCACAATAGAGAGAG	-	-
havcr1	GGGAGCATATGATGGACTGA	85%	0.92
hcst	GGCTAGCGTACCAGTAGGTGG	49%	0.93
il34	CCATGGTCCAGTCCGAATGC	77%	0.8
mrc1b	GCGCACCACAGACGCTGGTC	83%	0.85
tgm2l	GGGTCTCTACAGCATGACTG	92%	0.92
gpr183	GACTCTGTACTCAGCCAACC	86%	0.94
lgals3bp	GGTCTACCATGATGGACAGT	84%	0.88
sall3a	GGAGTGGATGATTCAGACAG	79%	0.91
smpd1	CGACGGGGATGTAGAGACGG	83%	0.91
tmem176.4	GGGTCATCAATATTGCATTG	31%	0.94
usp18	TATGTCCAGCAGTTCAGTTG	11%	0.97

Disease Models & Mechanisms • Supplementary information

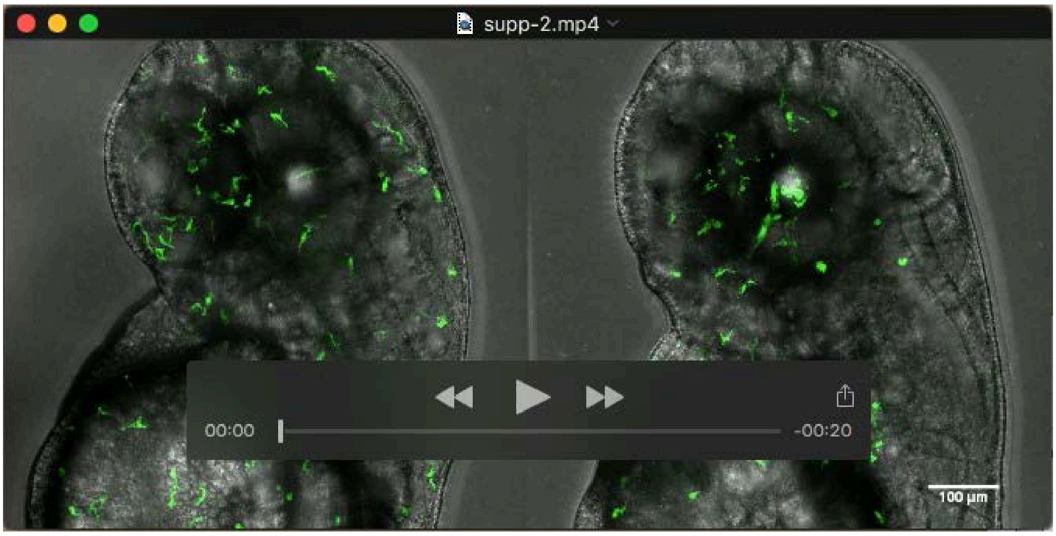

Movie 1. Timelapse imaging of a wildtype control (left) and *il34* mutant larva (right) between 2 and 3 dpf

Disease Models & Mechanisms • Supplementary information
